# Neuropsychiatric sequelae of long COVID-19: Pilot results from the COVID-19 neurological and molecular prospective cohort study in Georgia, USA

**DOI:** 10.1016/j.bbih.2022.100491

**Published:** 2022-07-18

**Authors:** Alex K. Chen, Xiaoling Wang, Lynnette P. McCluskey, John C. Morgan, Jeffrey A. Switzer, Rohini Mehta, Martha Tingen, Shaoyong Su, Ryan Alan Harris, David C. Hess, Elizabeth K. Rutkowski

**Affiliations:** a Medical College of Georgia at Augusta University, 1120 15th St, Augusta, GA, USA; b Georgia Prevention Institute, Medical College of Georgia at Augusta University, 1457 Walton Way, Augusta, GA, USA; c Department of Neuroscience and Regenerative Medicine, Medical College of Georgia at Augusta University, 1120 15th St, Augusta, GA, USA; d Movement and Memory Disorder Programs, Department of Neurology, Medical College of Georgia at Augusta University, 1120 15th St, Augusta, GA, USA; e Department of Neurology, Medical College of Georgia at Augusta University, 1120 15th St, Augusta, GA, USA; f Department of Psychiatry, Medical College of Georgia at Augusta University, 997 St. Sebastian Way, Augusta, GA, USA

**Keywords:** Long COVID, Mental health, Cognition, Cohort, Hyposmia, Coronavirus

## Abstract

**Background:**

As the coronavirus disease 2019 (COVID-19) pandemic continues, there has been a growing interest in the chronic sequelae of COVID-19. Neuropsychiatric symptoms are observed in the acute phase of infection, but there is a need for accurate characterization of how these symptoms evolve over time. Additionally, African American populations have been disproportionately affected by the COVID-19 pandemic. The COVID-19 Neurological and Molecular Prospective Cohort Study in Georgia (CONGA) was established to investigate the severity and chronicity of these neurologic findings over the five-year period following infection.

**Methods:**

The CONGA study aims to recruit COVID-19 positive adult patients in Georgia, United States from both the inpatient and outpatient setting, with 50% being African American. This paper reports our preliminary results from the baseline visits of the first 200 patients recruited who were on average 125 days since having a positive COVID-19 test. The demographics, self-reported symptoms, comorbidities, and quantitative measures of depression, anxiety, smell, taste, and cognition were analyzed. Cognitive measures were compared to demographically matched controls. Blood and mononuclear cells were drawn and stored for future analysis.

**Results:**

Fatigue was the most reported symptom in the study cohort (68.5%). Thirty percent of participants demonstrated hyposmia and 30% of participants demonstrated hypogeusia. Self-reported neurologic dysfunction did not correlate with dysfunction on quantitative neurologic testing. Additionally, self-reported symptoms and comorbidities were associated with depression and anxiety. The study cohort performed worse on cognitive measures compared to demographically matched controls, and African American patients scored lower compared to non-Hispanic White patients on all quantitative cognitive testing.

**Conclusion:**

Our results support the growing evidence that there are chronic neuropsychiatric symptoms following COVID-19 infection. Our results suggest that self-reported neurologic symptoms do not appear to correlate with associated quantitative dysfunction, emphasizing the importance of quantitative measurements in the complete assessment of deficits. Self-reported symptoms are associated with depression and anxiety. COVID-19 infection appears to be associated with worse performance on cognitive measures, though the disparity in score between African American patients and non-Hispanic White patients is likely largely due to psychosocial, physical health, and socioeconomic factors.

## Highlights

• Neuropsychiatric symptoms are often reported following COVID-19 infection.
• Self-reported symptoms may not be associated with objective dysfunction.
• Self-reported symptoms may be associated with depression and anxiety.
• Cognitive testing may overestimate clinical impairment in disadvantaged populations.

## 1 Introduction

Since it was first reported in the Chinese city of Wuhan in December 2019, coronavirus disease 2019 (COVID-19) has spread rapidly worldwide (Organization, 2021; Yüce et al., 2021). Preliminary data suggest that COVID-19 infection is highly associated with neurologic dysfunction involving the central nervous system (CNS) and peripheral nervous system (Almeria et al., 2020; Hampshire et al., 2021). While the precise mechanism behind such dysfunction has not been completely elucidated, SARS-CoV-2 is known to target the angiotensin-converting enzyme 2, which is present on neurons, endothelial cells, glial cells, and the choroid plexus and the subsequent neuroinflammation likely plays a role in the pathogenesis of such symptoms (Almutairi et al., 2021; Asadi-Pooya and Simani, 2020; de Erausquin et al., 2021; Huang et al., 2020; Matschke et al., 2020; Zhou et al., 2020).Acute neurological effects reported with COVID-19 include loss of taste, loss of smell, encephalopathy, and headache, with less common associations with seizures, ischemic stroke, and neuropathy (Almeria et al., 2020; Asadi-Pooya and Simani, 2020; Hampshire et al., 2021; Roy et al., 2021). Mood disorders have also increased in prevalence during the pandemic and are associated with increased susceptibility to COVID-19 infection as well as poorer outcomes (Ceban et al., 2021; Hao et al., 2020; Santomauro et al., 2021). There has been growing concern about the chronic neuropsychiatric sequelae of COVID-19 infection, including persistent hyposmia, cognitive impairment, fatigue, depression, insomnia, and anxiety, which in addition to other symptoms, has been termed “long COVID” or post-acute sequelae of SARS-CoV-2 infection (PASC) in the literature. (Ceban et al., 2022; Chou et al., 2021; Graham et al., 2021; Pavli et al., 2021; Phillips and Williams, 2021; Renaud-Charest et al., 2021).

Much of the current literature on the long-term neuropsychiatric sequelae of COVID-19 has relied on self-reported symptoms through questionnaires administered either in-person or through telephone interviews to comply with public health guidelines. However, the correlation between these self-reported symptoms and objective dysfunction is unclear. Self-reported depression has been demonstrated to overestimate clinical depression while self-reported symptoms such as anosmia and dysgeusia oftentimes underestimate the true prevalence of taste and smell dysfunction (Arias-de la Torre et al., 2020;<comment message=The citation(s) ‘de la Torre et al., 2020’ has/have been changed to match the author name/date in the reference list. Please check here and in subsequent occurrences, and correct if necessary.></comment> Yang and Pinto, 2016).

There is an obvious need for accurate characterization of the neuropsychiatric sequelae of COVID-19 and the risk factors associated with these outcomes. Such research should be cognizant of the potential disparities in how COVID-19 affects different populations. African Americans are estimated to be 13% of the United States population, yet they are 2.8 times more likely to be hospitalized and twice as likely to die from COVID-19 compared to White, non-Hispanic persons according to the Centers for Disease Control and Prevention (Mackey et al., 2021; Pennington et al., 2021). As the COVID-19 pandemic progresses, more patients will enter the chronic phase of the disease, and identification of groups at high risk of cognitive and psychiatric dysfunction may allow for targeted intervention to effectively meet their physical, neurological, and psychological health care needs. The COVID-19 Neurological and Molecular Prospective Cohort Study in Georgia (CONGA) study aims to recruit COVID-19 positive adult patients from the Central Savannah River Area (CSRA) of Georgia, United States with 50% being African Americans, representative of this community, to investigate the severity and chronicity of these neurologic sequelae and to assess for changes over the five-year period following infection. The objective of this paper is to discuss the preliminary findings from the first 200 patients in the CONGA study.

## 2 Methods

### 2.1 Study design

This is a single center, prospective, closed cohort study. We recruited adult patients who had tested positive for COVID-19 infection by respiratory swab or saliva sample reverse transcriptase polymerase chain reaction (RT-PCR) from the Central Savannah River Area (CSRA) of Georgia, where Augusta University (AU) Health System is a statewide COVID-19 testing site and has performed the majority of the testing for symptomatic and non-symptomatic individuals in the communities of CSRA. Recruitment began in March 2020, initially by contacting patients with a positive test through the AU Health testing services through telephone, and subsequently including a flier which allowed patients who were not tested through AU health to participate provided they submitted documentation of positive COVID-19 infection via testing by respiratory swab RT-PCR or saliva RT-PCR. Patients were evaluated at the clinical trials office at AU following their diagnosis with the goal of annual evaluations until five years after their baseline visit. This manuscript follows the Strengthening the Reporting of Observational Studies in Epidemiology (STROBE) guidelines.

### 2.2 Participants

CONGA Cohort: Inclusion criteria included patients testing positive for COVID-19 infection by respiratory swab or saliva sample RT-PCR. Enrolled patients were a minimum of four weeks from the date of confirmed COVID-19 infection or four weeks after the patient was discharged from the hospital. Both hospitalized and non-hospitalized patients were included. Hospitalized patients were tested upon admission. Participants had to be residents of the state of Georgia and older than 18 years old. Individuals with cognitive impairment and impaired decision-making capacity were included if consent was provided on their behalf by a legally authorized representative.

Patients were excluded if they were non-English speaking for whom a cognitive screen conducted in English would be an inaccurate representation of cognition.

Patients who met inclusion criteria were recruited via telephone call using an approved telephone script abbreviating details of the study and via community outreach efforts in COVID-19 education.

Control Cohort: The control cohort included participants who were enrolled in either the Georgia Cardiovascular Twin Study or the Georgia Stress and Heart study from 4/30/2019 to July 7, 2021. Criteria for classifying participants as African Americans or European Americans as well as study design and selection criteria have previously been described (Ge et al., 2006; Su et al., 2015). During the COVID-19 pandemic, a screening questionnaire was added to the visit protocol and COVID-19 positive patients have been documented and excluded from the current analysis. The institutional review board at the Medical College of Georgia gave approval for the studies. Each participant gave written consent prior to any involvement, in accordance with the institutional guidelines.

### 2.3 Procedures

Enrolled participants completed an initial questionnaire that included baseline characteristics such as age, sex, body mass index (BMI), highest level of education, and self-identified race (Supplemental Questionnaire). Participants also reported if they exercised, were hospitalized, had ever smoked tobacco products, were currently consuming alcohol, and had any medical comorbidities. The questionnaire also asked about symptoms attributed to acute COVID-19 infection with the option to write in other symptoms experienced. Patients also self-reported comorbid conditions with the option to write in any additional medical conditions.

Clinical measures included a full neurological examination including mental status, cranial nerves, motor function, sensory function, reflexes, coordination, and gait. Cognitive assessments included a Montreal Cognitive Assessment (MoCA) and NIH Toolbox (NIH-TB) for the Assessment of Neurological and Behavioral Function studies. NIH-TB studies included the tests of working memory (List Sorting Working Memory Test) and language (Picture Vocabulary Test). Scores were reported as age-adjusted national percentiles. Cognitive impairment was defined as a MoCA score less than or equal to 26 which is the cutoff for mild cognitive impairment (Nasreddine et al., 2005). NIH-TB scores were reported as national percentiles which are derived from the Toolbox census-weighted (for sex, education, and race/ethnicity) national norming study and are meant to act as a comparison to the general population. Individuals who scored less than the 25th national percentile were considered to have impairment in that domain.

A University of Pennsylvania Smell Identification Test (UPSIT) was to be completed at home. The UPSIT contains 40 multiple-choice questions involving the identification of microencapsulated odors to determine the degree of smell loss (Doty et al., 1989). A Waterless Empirical Taste Test (WETT) was also given to the participant to be completed at home, which includes 27 taste strips with responses (sweet, sour, bitter, salty, brothy, or no taste) to assess taste quality and concentrations (Doty et al., 2021). Once completed at home, both the UPSIT and WETT tests were scored by a researcher over the telephone or email. The UPSIT and WETT administration manuals provide interpretations of the score controlling for sex. A UPSIT score of ≤30 in females and ≤29 in males is defined as moderate hyposmia and this was used as the cutoff for quantitative evidence of changes in olfaction (Doty, 1995). A WETT score of ≤10 in males and ≤14 in females is defined as moderate taste loss and was used as the cutoff for quantitative evidence of changes in taste (Doty, 2020). Consented participants also had blood drawn by a research phlebotomist for future evaluation of serological, proteomic, genetic, metabolomic, and functional status related to COVID-19 infection and neurologic outcomes, such as oxidative stress and levels of inflammation. Plasma and peripheral blood mononuclear cells were frozen for later analysis.

Subjects completed mood questionnaires during their visit, including a Patient Health Questionnaire-9 (PHQ-9) for depression, which was defined as a score of 10 or higher, and a Generalized Anxiety Disorder-7 (GAD-7) screen for anxiety which was defined as a score of 8 or higher (Kroenke et al., 2001; Plummer et al., 2016).

### 2.4 Statistical methods

Descriptive statistics were provided for the demographic information and neuropsychiatric measures in our cohort. Spearman's rank correlation test was utilized to calculate the correlation among the neuropsychiatric measures. Correlation strength was defined as follows: 0.00–0.19 (very weak), 0.20–0.39 (weak), 0.40–0.59 (moderate), 0.60–0.79 (strong) and 0.80–1.00 (very strong) (Mukaka, 2012). Very weak correlations were not described in detail in this manuscript. Multivariate logistic regression was utilized to identify potential associations between neuropsychiatric measures and self-reported symptoms or comorbidities while controlling for age, sex, and ethnicity. Additionally, for cognitive measures including the MoCA and NIH-TB tests, we also controlled for education. For the analyses within the control cohort or the analysis between the control cohort and the CONGA study, generalized estimating equations (GEE) were used to account for family correlation (siblings and twins). P-values ≤ 0.01 were considered statistically significant. All analysis was done using STATA software.

### 2.5 Standard protocol approvals and patient consents

This study protocol was approved by the Institutional Review Board (IRB) at Augusta University Medical Center. Written consent detailing the purpose, procedures, data storage, risks, and benefits of the study was obtained at the initial visit. Given that we expected to encounter subjects with cognitive deficits during our study, patients completed a modified 10-question University of San Diego Brief Assessment of Capacity to Consent (UBACC) prior to providing informed consent independently with the option of consent provided by a legally authorized representative otherwise (Jeste et al., 2007).

## 4 Results

To date, over 20,000 cases in the CSRA have tested positive through our institution. At the time of analysis, 200 participants had been assessed at our clinical trials office. The demographic characteristics of the 200 participants included 71 males [35.5%] and a mean age of 44.6 years [Range 19–82]) (Table 1). 79 participants (39.5%) were African American, 14 participants (7%) were hospitalized for complications relating to acute COVID-19 infection, and the average time to first research visit was 125 days since positivity.

**Table 1:** Study participant demographics, self-reported symptoms, and comorbidities (n = 200).

Variables	Values
Age, years (mean (1 SD) [Min - Max])	44.59 (15.46) [19–82]
Education, years (mean (1 SD) [Min - Max])^a^	14.89 (1.99) [12–18]
BMI, kg/m^2^ (mean (1 SD) [Min - Max])^a^	31.74 (8.62) [19–71.9]
Days since diagnosis, days (mean (1 SD) [Min - Max])^a^	125.13 (48.64) [29–340]
Male, n (%)	71 (35.5)
Female, n (%)	129 (64.5)
Self-identified race	
Non-Hispanic White, n (%)	99 (44.5)
African American, n (%)	79 (39.5)
Other Race, n (%)	22 (11)
Exercise, n (%)^a^	141 (71.2)
Smoking, n (%)^a^	63 (31.7)
Alcohol, n (%)^a^	82 (41.2)
Comorbidity, n (%)^a^	154 (79.8)
Hospital, n (%)	14 (7)

a The numbers of available data for these variables are: 198, 188, 194, 198, 199, 199, and 193, respectively.

The prevalence of at least one self-reported neurological symptom attributed to COVID-19 was 160 out of 200. The most commonly reported COVID-19 symptom was fatigue (137 out of 200 patients [68.5%]), which was followed by headache (133 out of 200 [66.5%]) (Table 2). 109 out of 200 patients reported a change in smell (54.5%) and 108 out of 200 patients reported a change in taste (54%). 42 out of 200 patients reported confusion (21%). None of the patients in our cohort attributed stroke to their COVID-19 infection. A total of 59 unique comorbidities were reported by the first 200 participants. We excluded comorbidities that were reported in less than 5 participants for the analysis. Hypertension was the most common comorbid condition (81 out of 193 participants [42%]) (see Table 3).

**Table 2:** Self-reported symptoms (n = 200).

Symptom	n (%)
Fatigue	137 (68.5)
Headache	133 (66.5)
Muscle aches	114 (57)
Cough	111 (55.5)
Change smell	109 (54.5)
Change taste	108 (54)
Fever	100 (50)
Chills	96 (48)
Nasal congestion	95 (47.5)
Poor appetite	94 (47)
Shortness of breath	93 (46.5)
Runny nose	86 (43)
Sore throat	71 (35.5)
Diarrhea	70 (35)
Chest pain	59 (29.5)
Sneezing	59 (29.5)
Dizziness	54 (27)
Nausea	50 (25)
Confusion	42 (21)
Numbness	26 (13)
Vomiting	20 (10)
Vision change	20 (10)
Coordination problems	19 (9.5)
Dysarthria	8 (4)
Coma	1 (0.5)
Stroke	0 (0)

**Table 3:** Previous conditions (n = 193).

History of disease	n (%)
Hypertension	81 (42.0)
Hyperlipidemia	54 (28.0)
Obesity	42 (22.0)
History of depression	35 (18.1)
Diabetes	33 (17.1)
Reflux	33 (17.1)
Migraines	32 (16.6)
Arthritis	31 (16.1)
Sleep apnea	28 (14.5)
Chronic pain	22 (11.4)
Asthma	22 (11.4)
Gastritis	21 (10.9)
Cancer	19 (9.8)
Thyroid disease	19 (9.8)
Anemia	18 (9.3)
Insomnia	18 (9.3)
Coronary artery disease	11 (5.7)
Heart failure	9 (4.7)
Kidney disease	9 (4.7)
COPD/emphysema	6 (3.1)
Blood clots	5 (2.6)
History of stroke	5 (2.6)

### 4.1 Neuropsychiatric measurements

49 out of the 196 patients (25%) who completed a PHQ-9 met the criteria for depression (Table 4). 35 out of 195 participants (18%) who completed a GAD-7 met the criteria for anxiety. 89 of the 191 participants (47%) who completed a MoCA met the criteria for mild cognitive impairment. 196 participants completed the two NIH Toolbox tests. 58 participants (30%) demonstrated impaired vocabulary and 62 participants (32%) demonstrated impaired working memory. Age was negatively correlated with cognitive testing and was controlled for in all statistical analyses.

**Table 4:** Summary of neuropsychiatric measures.

Neuropsychiatric Quantitative measures	Value
PHQ-9 (n = 196), mean (1 SD)	6.12 (5.9)
GAD-7 (n = 195), mean (1 SD)	5.05 (5.5)
UPSIT (n = 164), mean (1 SD)	31.53 (5.2)
WETT (n = 159), mean (1 SD)	15.61 (5.4)
MOCA (n = 191), mean (1 SD)	25.17 (3.6)
NIH-TB language (n = 196), mean %ile (1 SD)	50.48 (30.5)
NIH-TB working memory (n = 196), mean %ile (1 SD)	43.76 (28.0)
**Depression, n (%)**	
Normal	102 (52.0)
Mild	45 (23.0)
Moderate	29 (14.8)
Moderate	12 (6.1)
Severe	8 (4.1)
**Anxiety, n (%)**	
Normal	111 (56.9)
Mild	49 (25.1)
Moderate	19 (9.7)
Severe	16 (8.2)
**Anosmia, n (%)**	
Normosmia	53 (32.3)
Mild hyposmia	62 (37.8)
Moderate hyposmia	32 (19.5)
Severe hyposmia	13 (7.9)
Anosmia	4 (2.4)
**Taste loss, n (%)**	
Normal	91 (57.2)
Mild loss	21 (13.2)
Moderate loss	25 (15.7)
Severe loss	22 (13.8)
**MoCA, n (%)**	
Normal (≥26)	102 (53.4)
Cognition impairment (<26)	89 (46.6)
**NIH-TB Language, n (%)**	
>25%	138 (70.4)
≤25%	58 (29.6)
**NIH-TB Working Memory, n (%)**	
>25%	134 (68.4)
≤25%	62 (31.6)

Out of the 164 subjects that had returned the UPSIT at the time of analysis, 49 (29.9%) had clinical evidence of impaired olfaction. Out of the 159 subjects that had returned the WETT at the time of analysis, 47 (29.6%) had clinical evidence of impaired taste. Age, sex, and ethnicity were not significantly associated with smell or taste impairment.

### 4.2 Correlation among neuropsychiatric outcomes

A Spearman rank correlation was performed among the neuropsychiatric measurements (Table 5). Strong correlations were found between PHQ-9 and GAD-7 score (r = 0.66, p < 0.0001) as well as between NIH-TB language and MoCA score (r = 0.62, p < 0.0001). Moderate correlations were found between NIH-TB working memory and MoCA score (r = 0.48, p < 0.0001) and NIH-TB language (r = 0.49, p < 0.0001). Weak correlations were observed for UPSIT with WETT (r = 0.26, p = 0.0016), MoCA (r = 0.30, p = 0.0002), and NIH-TB language (r = 0.36, p < 0.0001).

**Table 5:** Correlations among neuropsychiatric measures using Spearman's Rank correlation test.

	PHQ-9	GAD-7	WETT	UPSIT	MoCA	NIH-TB Language	NIH-TB Working Memory
PHQ-9	1						
GAD-7	**0.66**	1					
WETT	0.01	0.07	1				
UPSIT	−0.04	0.11	**0.26**	1			
MOCA	**−**0.17	0.04	0.15	**0.30**	1		
NIH-TB Language	−0.12	0.011	0.18	**0.36**	**0.61**	1	
NIH-TB Working Memory	−0.13	−0.14	0.00	0.17	**0.48**	**0.49**	1

Values reported are correlation coefficients and values with p < 0.01 are bolded.

### 4.3 Relationships between reported symptoms and associated objective measures

There were no significant associations between self-reported hyposmia and hypogeusia attributed to COVID-19 and their associated quantitative measures. The percentage of patients with abnormal MoCA and NIH-TB scores did not significantly differ between patients endorsing cognitive symptoms compared to those who did not.

### 4.4 Relationships between neuropsychiatric measures and other symptoms and comorbidities

There were many significant associations between self-reported symptoms significantly associated with depression and anxiety (Table 6). The summary of the significant associations between neuropsychiatric measures and self-reported symptoms is summarized in a supplemental table (Supplemental Table 1). Diabetes, obesity, sleep apnea, and a history of depression were significantly associated with objective measures of depression. Anemia and a history of depression were significantly associated with an increased likelihood of objective anxiety.

**Table 6:** Prevalence of depression among patients with self-reported symptoms compared to patients not endorsing symptoms.

a			
Self - reported Symptom	Depression prevalence in those reporting symptom	Depression prevalence those not reporting symptom	p-value
Shortness of breath	42.9%	9.6%	<0.001
Chest pain	50.9%	14.0%	<0.001
Fatigue	30.8%	12.9%	0.01
Nausea	46.9%	17.8%	<0.001
Diarrhea	38.8%	18.0%	0.003
Confusion	56.1%	16.9%	<0.001
Dizziness	43.4%	18.3%	0.002
Numbness	53.9%	20.7%	0.001
Change in vision	60.0%	21.1%	<0.001
Coordination problems	55.6%	22.0%	0.007
b			
	Anxiety prevalence in those reporting symptom	Anxiety prevalence in those not reporting symptom	p-value
Cough	24.8%	9.3%	0.004
Chest pain	33.9%	11.0%	0.002
Change in taste	25.2%	9.1%	0.008
Confusion	34.2%	13.6%	0.005
Numbness	34.6%	15.4%	0.007

### 4.5 Comparison to control cohort

342 participants were enrolled in either the Georgia Cardiovascular Twin Study (n = 243, 97 twin pairs and 49 singletons) or the Georgia Stress and Heart study (n = 112) from 4/30/2019 to July 7, 2021. The general characteristics of the control cohort and CONGA cohort patients who completed the NIH-TB tests are summarized (Table 7). The average age of the control cohort (35.8 ± 5.3 years) was lower than the average age of the CONGA cohort (45.4 ± 15.5 years) therefore the analyses were repeated in the CONGA cohort for patients that were under the age of 50.

**Table 7:** General characteristics of the control cohort.

	Control cohort	CONGA^a^	CONGA with age ≤ 50^a^
N	342	178	110
Age, mean (1 SD) [min - max]	35.8 (5.3) [19.4–46.5]	45.4 (15.5) [19–82]	35.2 (1.8) [19–50]
Male, %	37.1	37.1	39
AA, %	58.8	44.4	40.9
Education, %			
High school	20.8	19.8	16.5
Some college	38.1	36.2	36.7
College	27.0	25.4	32.1
Post-college	14.1	18.6	14.7
NIH-TB Working Memory Score, %ile (SD)	52.8 (30.7)	43.9 (28.2)	43.4 (28.6)
NIH-TB Working Memory, % with <25%	27.73	30.86	30.91

a Only participants with self-identified race as either Non-Hispanic White or African American were included.

Compared to the control cohort, the average percentile on the NIH-TB working memory test in our study was significantly lower in both the general cohort and in patients under the age of 50 (Whole cohort: 52.3 vs 43.9; p < 0.001 and under 50: 52.3 vs 43.4; p < 0.001). However, the percentage of patients with impaired working memory in the control cohort compared to the CONGA cohort was not statistically significant (Whole cohort: 28.32% vs 30.86%; p = 0.107 and under 50: 28.32% vs 30.91%, p = 0.138).

African American participants performed worse in all cognitive tests compared to White, non-Hispanic participants after controlling for age, sex, and education (Table 8).

**Table 8:** Ethnic differences in cognitive measurements between Non-Hispanic White and African American participants.

Measurement	Non-Hispanic White	African American	p^a^
Quantitative, mean (1 SD)			
MoCA	26.77 (2.47)	23.21 (3.51)	<0.001
NIH-TB language	67.82 (24.67)	30.26 (24.42)	<0.001
NIH-TB working memory	54.62 (25.98)	30.52 (24.96)	<0.001
Categorical, n (%)			
MoCA	22 (23.4)	57 (75)	<0.001
NIH-TB language	8 (8.24)	43 (55.12)	<0.001
NIH-TB working memory	14 (14.43)	40 (51.28)	<0.001

a Age, sex and education level were adjusted.

## 5 Discussion

### 5.1 Key results

We analyzed the preliminary data from the baseline visits of the first 200 patients enrolled in the CONGA study. Novel findings include evidence that self-reported symptoms may not correlate with quantitative testing. These data underscore the importance of quantitative testing in the accurate assessment of deficits. African American patients appear to score significantly worse on quantitative cognitive testing compared to Non-Hispanic White patients, which likely underscore the disparities in how cognitive tests assess different ethnic groups due to various systemic factors including differences is socioeconomic status, psychosocial factors, and physical health. Findings of the present investigation reinforce the evidence from previous studies that patients who have been infected with COVID-19 continue to report neuropsychiatric effects beyond the acute phase of infection, which has been termed “long COVID” or PASC. We found evidence of worsened cognitive performance in our cohort compared to the control group though it did not meet our threshold for clinical significance.

### 5.2 Self-reported symptoms of COVID-19 are not predictive of objective long-term dysfunction

Self-reported change in smell is an established predictor of acute COVID-19 infection (Gerkin et al., 2020). Yet, while the majority of our participants reported changes in olfaction and taste due to COVID-19 (54.5% and 54% respectively), there was no significant association between these reported symptoms and the quantitative measures of anosmia and dysgeusia.

In fact, a higher proportion of clinical anosmia and dysgeusia was noted in patients who did not report a change in smell and taste. There are multiple explanations for why those who self-report olfactory and gustatory changes may not present with evidence of objective abnormalities on clinical testing. It is possible that participants recovered those senses following acute infection as the mean time from positive COVID-19 test to clinical visit in our study was 125 days. A recent review of olfactory dysfunction following COVID-19 infection found that resolution of self-reported symptoms occurs an average of 10 days after symptom onset (Xydakis et al., 2021). Additionally, the higher prevalence of self-reported olfactory and gustatory deficits compared to quantitative testing highlights how the change in smell and taste associated with COVID-19 may manifest as qualitative changes. Such changes may include parosmias such as dysosmias and phantosmias rather than simply the absence of these senses, which may not be as readily apparent with our quantitative tests (Hummel et al., 2011). This may explain why those who still self-reported persistent smell and taste abnormalities still scored within the normal range on the UPSIT and WETT.

“Brain fog” is a colloquial term utilized to describe the constellation of cognitive symptoms following COVID-19 infection and has been considered a significant and debilitating aspect of long-COVID (Graham et al., 2021). However, in our study, patients who reported cognitive symptoms such as confusion and fatigue were not more likely to have cognitive deficits on quantitative cognitive testing compared to those who did not report cognitive symptoms after controlling for age, sex, ethnicity, and education. Given the high prevalence of depression after COVID-19 infection related to both the psychosocial effects of the COVID-19 pandemic as well as the implications of the virus’ long-term effects, it is possible that a proportion of patients endorsing subjective cognitive impairment may have other underlying psychiatric factors which may be contributing towards brain fog symptoms.

### 5.3 Self-reported symptoms are associated with psychiatric dysfunction

In the present investigation, 80% of patients reported neurological symptoms. The most common reported COVID-19 symptom was fatigue (137 out of 200 patients [68.5%]) followed closely by headache (133 out of 200 [66.5%]) which is similar to what has been previously reported (Nalbandian et al., 2021). Patients who self-reported symptoms were more likely to have persistent depression and anxiety. A recent meta-analysis concluded that there has been an increase in the prevalence of major depressive disorder and anxiety disorders during the COVID-19 pandemic associated with increased rates of SARS-CoV-2 infection and decreased mobility (Santomauro et al., 2021). As depression was already the leading cause of disability worldwide prior to the COVID-19 pandemic, the increase of depressive and anxiety disorders during the pandemic highlights the importance of strengthening mental health institutions.

### 5.4 Patients may experience cognitive impairment beyond acute COVID-19 infection

Compared to our control cohort, the CONGA cohort scored significantly lower on the NIH-TB working memory test based on age-adjusted national percentiles. Previous data published with NIH-TB testing in COVID-19 patients also found that working memory was impaired in COVID-19 patients compared to the general population based on demographic-matched normative scores (Graham et al., 2021). Notably their cohort was comprised of only 6% African Americans while our cohort was comprised of 40% African American of whom may be at higher risk of a more severe clinical course following infection, disparities of which may persist beyond acute infection (Kullar et al., 2020; Mackey et al., 2021; Yancy, 2020). In the present investigation, African American patients scored significantly lower on quantitative cognitive testing (MoCA and NIH-TB tests) compared to Non-Hispanic White patients. After controlling for age, sex, and education, African American patients were significantly more likely to meet our criteria for mild cognitive impairment (75% in African Americans vs 23.4% in Non-Hispanic Whites; p < 0.001). The significantly lower cognitive scores may reflect yet another domain that may contribute to the health disparities observed in COVID-19 infection. The extent to which this discrepancy is attributable to COVID-19 infection is unclear as such a difference was similarly observed in our control cohort. In fact, previous literature has also suggested that in African Americans, the traditional normative cutoff of 26 on the MoCA for mild cognitive impairment may not be as reflective of true impairment as scores in this range may be asymptomatic (Rossetti et al., 2017). African American patients in our cohort had an average MoCA of 23.2 which is similar to what has been previously proposed in the literature as a possible cutoff for mild cognitive impairment. The cause for this discrepancy is likely multifactorial, and is likely related to structural disparities involving psychosocial factors such as experienced racism, physical health including a higher rate of comorbidities, and socioeconomic factors such as educational resources, wealth, nutrition, and environmental exposures (Chin et al., 2012; Coogan et al., 2020; Rosso et al., 2016; Zahodne et al., 2017). Such a difference underscores the continued need for cognitive screens such as the MoCA to account for cultural and ethnic differences to avoid overestimating, diagnosing, and treating cognitive impairment in select populations.

### 5.5 Olfactory dysfunction persists beyond acute COVID-19 infection

The prevalence of persistent objective smell and taste dysfunction (30% and 30% respectively) in our cohort is well within the range of what has been previously reported via subjective measures and quantitative olfactometry in the literature (Nalbandian et al., 2021; Renaud et al., 2021; Xydakis et al., 2021). Previous studies have demonstrated that age and sex are the most predictive of smell dysfunction in the general population, with increasing age and male sex being associated with a higher prevalence of olfactory dysfunction (Liu et al., 2016; Yang and Pinto, 2016). The age and sex of our patients were well within the range of previous studies, with the mean age of our patients (45 ± 15 years) being lower than the average or median age of previous studies (43.7–70.5), and our percentage of male patients (35.5%) being similar as well (Nalbandian et al., 2021; Renaud et al., 2021; Xydakis et al., 2021).The prevalence of objective smell and taste dysfunction is higher than what has been reported in previous population-based studies prior to the COVID-19 pandemic, which estimate the prevalence of smell dysfunction to be 2.7–24.5% and the prevalence of taste dysfunction to be 17.3% (Liu et al., 2016; Yang and Pinto, 2016). These data suggest that there is persistent loss of olfactory and gustatory sensation following COVID-19 infection. Whether these deficits are associated with abnormal cortical function has not been definitively demonstrated. A fluorodeoxyglucose (FDG)-positron emission tomography (PET) study of 12 COVID-19 patients with acute hyposmia demonstrated abnormal glucose metabolism within the olfactory tract and frontal lobe; however, a separate study of 12 COVID-19 patients with abnormal olfaction found no evidence of abnormal frontotemporal function on functional near-infrared spectroscopy (fNIRS). (Ho et al., 2021; Niesen et al., 2021). While these results are limited due to their small sample size and lack of premorbid comparison, results published utilizing the United Kingdom biobank demonstrated generalized cortical atrophy, especially in the orbitofrontal cortex, parahippocampal gyrus, and primary olfactory cortex when compared to controls. This study utilized the magnetic resonance imaging (MRI) data of 785 participants, of which 401 mild cases of COVID-19. Notably, these participants all had MRI images prior to the onset of the pandemic which was utilized for comparison (Douaud et al., 2022).

### 5.6 Future directions

With many patients reporting symptoms beyond the acute phase of COVID-19, there is a necessity for effective treatments. One intervention which has demonstrated effectiveness for psychiatric symptoms is cognitive behavioral therapy (CBT) (Ho et al., 2020). Specifically, internet CBT has been demonstrated to be a cost-effective method for many psychiatric conditions while adhering to public health guidelines (Soh et al., 2020; Zhang and Ho, 2017). Additionally, there is an interest in whether COVID-19 may increase the risk of developing dementia (de Erausquin et al., 2021). Symptoms commonly associated with COVID-19, such as anosmia, have previously been associated with the onset of dementia and neurodegeneration (Quarmley et al., 2017; Schrag et al., 2017). The pandemic may provide the opportunity to investigate the mechanism behind the association between anosmia and dementia. In the future, we plan to analyze serum and mononuclear cells drawn from patients as these markers have already shown promise as a predictor of disease severity (Loomba et al., 2021; Manunta et al., 2021). As we continue to recruit participants and ensure long-term follow-up, we anticipate that predictors of neurological dysfunction will become clearer.

### 5.7 Strengths and limitations

Strengths of our study include the large sample size, the unique racial diversity of our community, and the use of validated quantitative measurements of smell, taste, and cognitive function. Limitations to this study include the inability to administer smell and taste tests in the clinic setting to follow public masking health guidelines resulting in a lower yield of returned test results. Given the high prevalence of COVID-19 during the pandemic, it is impossible to rule out exposure to COVID-19 in the control group, though we attempted to minimize this effect by excluding patients utilizing the screening questionnaire. Additionally, patients who seek out SARS-CoV-2 testing may have intrinsic differences than those that do not, such as the motivation and health literacy to seek out tests.

### 5.8 Generalizability

We feel that our study should be generalizable to non-hospitalized COVID-19 patients while understanding that geographic and demographic differences may affect outcomes. Given the relatively low percentage of hospitalized patients (7%), this study may not be representative of the outcomes of hospitalized patients.

## Declaration of competing interest

D.C.H. receives funding from the National Institute of Neurological Disorders and Stroke (NIH/NINDS R01NS112511-01A1S1 (Hess, D)). The remaining authors declare that they have no known competing financial interests or personal relationships that could appear to influence the work in this paper.

David Hess reports financial support was provided by 10.13039/100000065National Institute of Neurological Disorders and Stroke. David Hess reports financial support was provided by TR Reddy Family Fund.

## Data Availability

Data will be made available on request.
