# Supplementary material for: Neuropsychiatric sequelae of long COVID-19: Pilot results from the COVID-19 neurological and molecular prospective cohort study in Georgia, USA
Source: Brain Behav Immun Health. 2022 Jul 18;24:100491. doi: 10.1016/j.bbih.2022.100491 (PMC9290328; doi:10.1016/j.bbih.2022.100491)
Supplement: Multimedia component 1 [file mmc1.pdf]

Subject: ____

(CONGA) COVID-19 Neurological and Molecular Prospective Cohort Study in Georgia

Subject's Personal and Health/Medical Information

Date:
**Contact Information**
Last name:
First name:
Cell phone:
May we contact you via text messages for study updates and reminders? ☐ Yes ☐ No
E-mail address (please print clearly):
May we contact you via e-mail for study updates and reminders? ☐ Yes ☐ No
Mailing address (please print clearly): ☐ This is different from my home address
May we mail you study-related documents and test kits to this address? ☐ Yes ☐ No
**Personal Information**
Date of birth:
Sex: ☐ M ☐ F ☐ other
Race/ethnicity:<br>☐ White<br>☐ Hispanic, Latino, or Spanish<br>☐ Black or African<br>☐ Asian<br>☐ Pacific Islander<br>☐ Middle Eastern<br>☐ American Indian<br>☐ Mixed Race<br>☐ Prefer not to answer<br>☐ Other: ______________________
SSN:

Marital status: ☐ Single ☐ Married ☐ Divorced ☐ Widowed

<u>Highest level of education</u> **(Yourself)**:	<u>Highest level of education</u> **(Your Spouse)**:
☐ Less than high school	☐ Less than high school
☐ High school or equivalent	☐ High school or equivalent
☐ Vocational/technical school	☐ Vocational/technical school
☐ Some college	☐ Some college
☐ Associate's degree	☐ Associate's degree
☐Bachelor's degree	☐ Bachelor's degree
☐ Master's degree	☐ Master's degree
☐ Professional degree	☐ Professional degree
☐ Doctorate degree	☐ Doctorate degree
Occupation **(Yourself):**	Occupation **(Your Spouse)**:

Emergency Contact

First and last name:

Phone number:

Relationship:

Medical History

Personal medical history (check if yes):

High blood pressure		Gastritis/reflux	
Diabetes		Dementia	
High cholesterol		Stroke	
Asthma		Epilepsy/seizures	
COPD/emphysema		HIV/AIDS	
Heart failure		Arthritis	
Coronary artery disease		Chronic pain	
Heart arrhythmia		Insomnia	
Thyroid disease		Obesity	
Cancer		Migraines	
Depression		Anemia	
Kidney disease			
Liver disease			
Sleep apnea			
Blood clots			

Others: ____

____

Medications (please list all medications you are currently taking, including prescription, over-the-counter, vitamins and supplements):

Medication	Dose

Allergies:

Exercise: Do you exercise? ☐ Yes ☐ No

If yes, describe type of exercise: ______________________________

How frequent? ______________________________

Intensity: ☐ low ☐ moderate ☐ hard

Tobacco: Have you ever smoked? ☐ Yes ☐ No

If yes, Circle one: cigarettes cigar vape/e-cigarette
How much currently? __________________________
If prior smoker, when did you quit? _______________

Alcohol: ☐ Yes: _____ drinks per week / month (circle one) ☐ No

Recreational substances: ☐ Yes (list): ___________________________ ☐ No

Family history (please list any medical problems that run in your family):

COVID-19 symptoms you experienced (check if yes):

Fever		Change in smell	
Chills		Change in taste	
Cough		Headache	
Shortness of breath		Confusion	
Chest pain/pressure		Coma	
Muscle aches		Dizziness	
Runny nose		Numbness or tingling	
Sore throat		Seizure	
Sneezing		Stroke	
Nasal congestion		Difficulty speaking	
Poor appetite		Vision changes	
Fatigue		Coordination problem	
Nausea		Other: (please list)	
Vomiting			
Diarrhea			
