# Supplementary material for: Neuropsychiatric sequelae of long COVID-19: Pilot results from the COVID-19 neurological and molecular prospective cohort study in Georgia, USA
Source: Brain Behav Immun Health. 2022 Jul 18;24:100491. doi: 10.1016/j.bbih.2022.100491 (PMC9290328; doi:10.1016/j.bbih.2022.100491)
Supplement: Multimedia component 2 [file mmc2.docx]

**Supplemental table 1a.** Significant associations between depression and self-reported comorbidities

| **Self-reported Comorbidity** | **Depression prevalence in those reporting comorbidity** | **Depression prevalence in those not reporting comorbidity** | **p-value** |
| --- | --- | --- | --- |
| Diabetes | 40.6% | 22.1% | 0.008 |
| Obesity | 46.3% | 19.5% | <0.001 |
| Sleep apnea | 42.9% | 22.2% | 0.007 |
| History of depression | 68.6% | 15.6% | <0.001 |

**Supplemental Table 1b.** Significant associations between anxiety and self-reported comorbidities

| **Self-reported Comorbidity** | **Anxiety prevalence in those reporting comorbidity** | **Anxiety prevalence in those not reporting comorbidity** | **p-value** |
| --- | --- | --- | --- |
| Diabetes | 40.6% | 22.1% | 0.008 |
| Obesity | 46.3% | 19.5% | <0.001 |
| Sleep apnea | 42.9% | 22.2% | 0.007 |
| History of depression | 68.6% | 15.6% | <0.001 |
| Anemia | 52.9% | 14.6% | 0.002 |
| History of depression | 37.1% | 13.8% | 0.007 |
